# Development and characterization of specific anti‐Usutu virus chicken‐derived single chain variable fragment antibodies

**DOI:** 10.1002/pro.3937

**Published:** 2020-09-04

**Authors:** Amelie Karin Josephine Schoenenwald, Chin Piaw Gwee, Karin Stiasny, Marcela Hermann, Subhash G. Vasudevan, Tim Skern

**Affiliations:** ^1^ Max Perutz Labs Medical University of Vienna, Vienna Biocenter Vienna Austria; ^2^ Programme in Emerging Infectious Diseases Duke‐NUS Medical School Singapore; ^3^ Center for Virology Medical University of Vienna Vienna Austria; ^4^ Department of Microbiology and Immunology National University of Singapore Singapore; ^5^ Institute for Glycomics Griffith University, Gold Coast Campus Queensland Australia

**Keywords:** antigenic cross‐reaction, DIII, envelope protein, flavivirus, flavivirus diagnostic, phage display, scFv, Usutu virus, West Nile virus

## Abstract

Usutu virus belongs to the Japanese encephalitis serogroup within the *Flaviviridae* family. Mammals may become incidental hosts after the bite of an infected mosquito while birds act as the main reservoir. Human cases have become more common recently and elicit various outcomes ranging from asymptomatic to severe illness including encephalitis. Problematically, antisera against Usutu virus cross‐react with other flaviviruses such as the co‐circulating West Nile virus. As an approach to generate Usutu virus‐specific antibodies, we immunized chickens with purified Usutu virus envelope protein domain III, isolated the spleen mRNA and generated an scFv phage display library. The most potent binders for Usutu virus domain III were selected via biopanning and their affinity to domain III was examined using SPR. Four scFvs bound the domain III of Usutu virus in the nanomolar region; two bound the protein over 40 times more strongly than West Nile virus domain III. We further characterized these scFv antibodies for suitability in standard laboratory tests such as western blots, ELISA, and neutralization tests. Four specific and one cross‐reactive antibody performed well in western blots with domain III and the full‐length envelope protein of Usutu virus and West Nile virus. All antibodies bound in virus ELISA assays to Usutu virus strain Vienna‐2001. However, none of the antibodies neutralized either Usutu virus or West Nile virus. These antibody candidates could be crucial in future diagnostic tests to distinguish Usutu virus from other flaviviruses and might even offer virus neutralization after a conversion to Fab or IgG.

## 1 INTRODUCTION

The genus *Flavivirus* is composed of over 70 members such as Zika virus (ZIKV), Dengue virus (DENV), and Japanese encephalitis virus (JEV). Flaviviruses are spherical, enveloped viruses with a diameter of 40–60 nm and a positive sense, single‐stranded RNA genome of about 11 kb length with a type I 5′ cap and no poly‐A tail.^1^ Both Usutu virus (USUV) and West Nile virus (WNV) are mosquito‐borne flaviviruses belonging to the JEV serocomplex.^1^, ^2^, ^3^ Originating from Africa, USUV has recently emerged in Europe after its introduction to Italy by migratory birds in 1996.^4^ It propagates via a zoonotic life cycle with birds as amplifying hosts and ornithophilic mosquitoes acting as vectors.

To date, 93 human cases of serological evidence for USUV and 53 acute infections have been described out of which most progressed asymptomatically.^5^, ^6^, ^7^ However, in some cases mild influenza‐like symptoms were associated with USUV infection, but more severe courses with invasion of the central nervous system in immunocompromised patients,^8^, ^9^, ^10^ meningoencephalitis and meningitis,^9^, ^11^, ^12^, ^13^, ^14^, ^15^ and sudden facial paralysis in a healthy 39‐year‐old man have also been described.^16^

Generally, human cases of USUV infections are sporadic but the number has increased, especially since comprehensive WNV surveillance has been implemented.^17^ USUV and WNV co‐circulate^18^ and share overlapping transmission cycles.^19^ These circumstances, along with extensive cross‐reactivity among flaviviruses owing to sequence similarity in the envelope (E) protein, might have led to misinterpretation of serological test results.^20^, ^21^, ^22^ Indeed, WNV and USUV have been misdiagnosed for each other^23^ and several infections were falsely attributed to WNV before thorough genetic re‐evaluation.^24^, ^25^ The high cross‐reactivity found in antibodies is still a challenge for differential diagnosis of infections by USUV and WNV.^26^, ^27^ Further, such cross‐reactivity has been linked to severe sickness via antibody‐dependent enhancement in sequential infections of different flaviviruses.^25^ The cross‐reactivity among flaviviruses, especially between the co‐circulating WNV and USUV, and the current lack of reliable commercial tests for USUV may have resulted into a vastly underestimated presence of USUV in Europe. Its low prevalence in the human population bears the risk of a potential outbreak in Europe.^6^, ^11^

Thus, we strived to develop single chain variable fragment (scFv) antibodies that are specific for USUV. These scFvs presented the first step towards the development of a diagnostic tool for USUV. We chose scFv over Fab fragments because they could easily be integrated in a phage display protocol as scFvs have a higher stability for panning while their single peptide character allows for bacterial expression in a functional form. Furthermore, optimization of affinity and specificity is cost‐efficient.^28^ Moreover, scFv have been shown to provide better pharmacokinetic features than mAbs or IgGs, stressing their potential benefits for diagnostic applications.^29^

Here, we aimed at closing the diagnostic gap for USUV infections by generating, with the assistance of phage display technology, USUV‐specific scFv antibodies that do not cross‐react with WNV. We describe here the isolation and characterization of four scFv antibodies derived from a chicken library that recognize the domain III (DIII) of the USUV E protein with nanomolar affinities and up to a 50 fold higher specificity to USUV than WNV as well as on antibody that recognizes the E protein DIII of both USUV and WNV with similar affinities.

## 2 RESULTS

### 2.1 *Generation of an scFv chicken library against USUV DIII*

Given our previous inability to generate soluble scFv derived from the E16 antibody (manuscript submitted), we decided to take an alternative approach to generate scFv specific for USUV that would be derived from chicken IgY antibodies. To this end, we first immunized three chickens against group A and group I USUV DIII using the protocols indicated in Methods. Isolated IgY from all three chickens showed a clear increase in immune reactivity towards the antigens with which they were immunized but also to DIII from WNV (Fig. S1). After RNA extraction from each chicken spleen, cDNA synthesis and PCR amplification of the *V*_H_ and *V*_λ_ fragments, the respective fragments from all three chickens were pooled for subsequent overlap extension PCR to link them together and to further expand the library. The resulting library had a size of 1.5 × 10^7^ transformants and was subsequently expanded to 3.3 × 10^13^ transformants per ml by bacterial amplification of the phage to increase the probability of complete coverage.

### 2.2 *Solid‐phase panning on USUV DIII*

**FIGURE 1:**
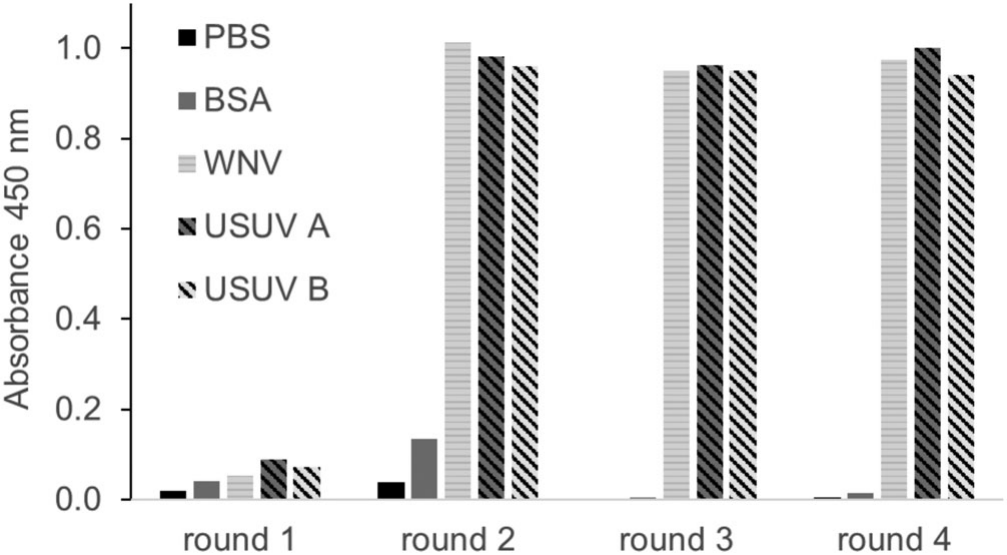
Enrichment of bacteriophage expressing E protein DIII during panning. As an antigen, USUV group A and group B were coated. Background binding was evaluated by including PBS and BSA while specificity for USUV was monitored by including WNV E protein DIII (*n* = 1). A clear enrichment already after the second panning was observed

**FIGURE 2:**
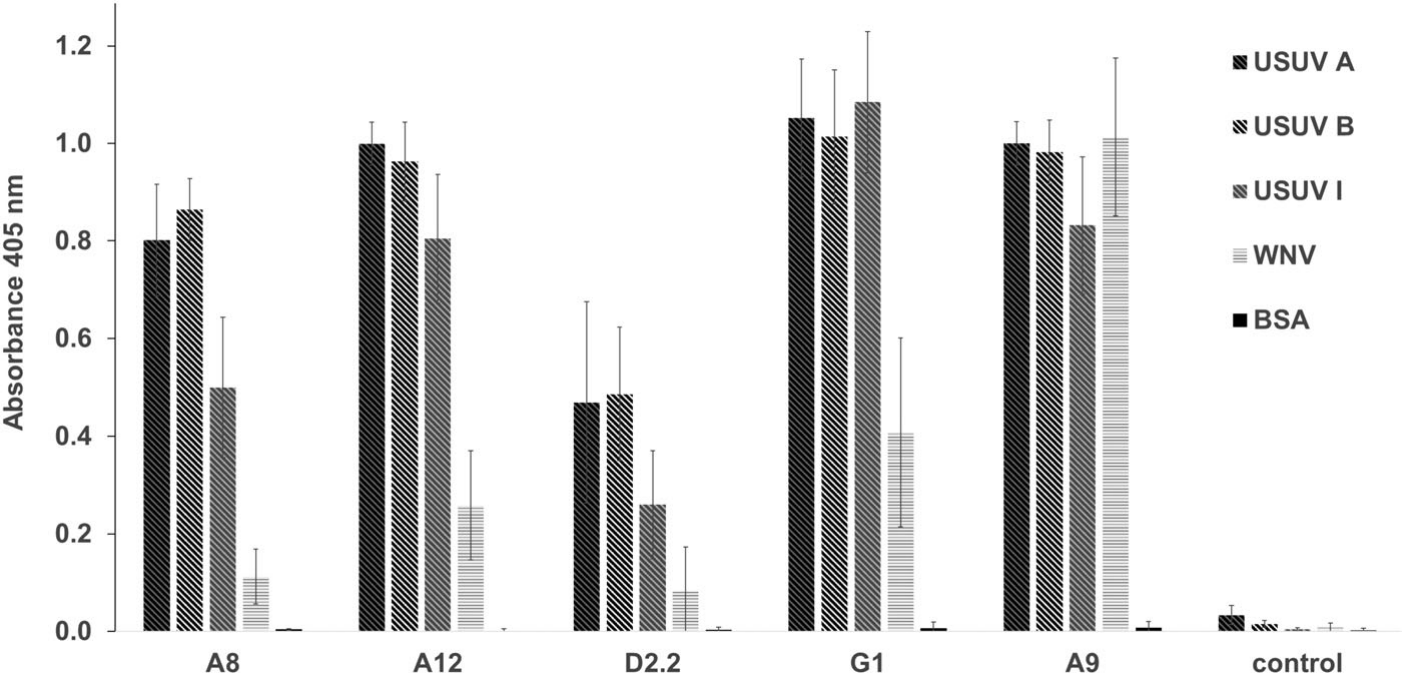
Binding of scFv to E protein DIII antigens in an ELISA. The E protein DIII antigens of USUV A, B, I and WNV, were coated on high‐binding ELISA plates. BSA in PBS was used as a negative control. The plates were incubated with the indicated scFvs, followed by mouse anti‐HA antibodies, and finally HRP‐conjugated goat anti‐mouse antibodies for visualization. The results were obtained from at least four independent experiments each with triplicates. The error bars represent the standard error

The DIII‐immune phage display scFv library was panned four times alternatingly against USUV A and USUV B with increasing stringency using high‐absorbing ELISA plates. Polyclonal ELISAs were performed to monitor the enrichment of specific scFv in each round of panning against USUV E protein DIII. Figure 1 shows the initially low background binding towards PBS and BSA from the first two rounds being eliminated from round three onwards; however, enrichment towards the flaviviral DIIIs occurred already in the second panning. Then, 380 randomly picked clones from rounds 2, 3, and 4 were cultivated and the binding specificity of single clones was evaluated in monoclonal ELISAs (data not shown). Clones were considered specific for USUV antigen when their OD_405_ signals were at least three‐fold higher compared to WNV E protein DIII. This revealed 34 USUV E protein DIII‐specific clones out of which 26 were identified as being unique clones via BstNI fingerprinting (Fig. S2) and subsequent Sanger sequencing. The scFv antibodies produced by these clones were screened for specificity against DIII of WNV and USUV group A, B, and I (manuscript submitted) using ELISA. The four scFvs with the strongest signal for USUV and one scFv that was shown to be specific for WNV were chosen for further examination (see Figure 2). The results showed that the third panning round was very stringent, as there were only few clones on the probing plate for monoclonal ELISA. Consequently, the clones that were amplified in the fourth round were mostly identical and the identified antibodies are highly cross‐reactive with WNV. Interestingly, the four USUV‐specific and the cross‐reactive scFv were all recovered from the second round of panning.

### 2.3 *Characterization of selected scFv antibodies against USUV DIII*

**FIGURE 3:**
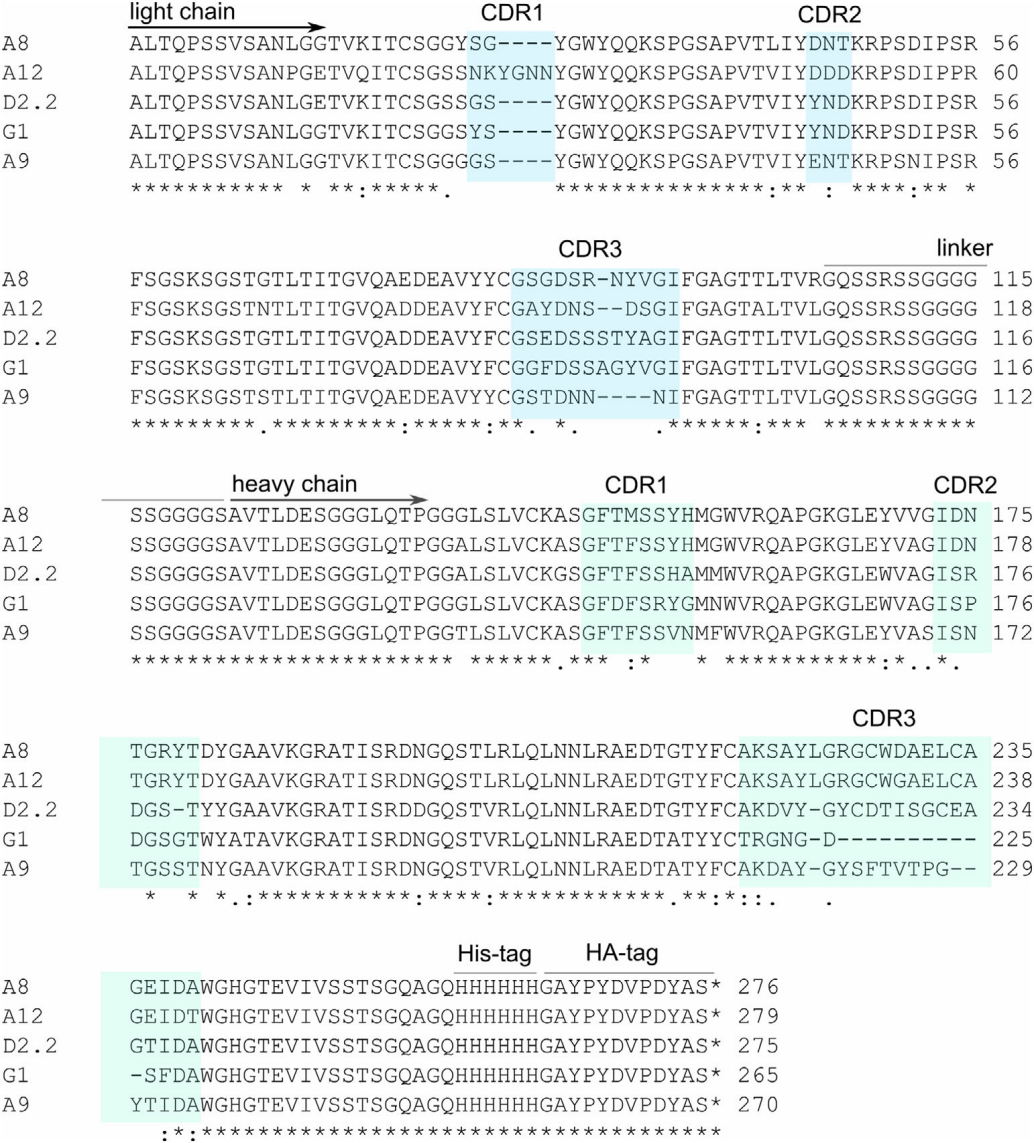
Multiple sequence alignment of five scFv antibodies. A multiple amino acid sequence alignment was performed with Clustal Omega.^38^ The CDR were identified using IMGT/V‐QUEST.^40^, ^41^ The N‐terminal light chain of the scFvs comprises three CDRs and is connected to the heavy chain and its three CDRs via a linker. All scFvs carry a C‐terminal His‐tag and HA‐tag for purification and detection. A considerable variation in amino acid sequences (amino acid substitutions, insertions, and deletions) can be seen in all CDRs

Based on the ELISA panning examination (Figure 2), four scFvs (A8, A12, D2.2, and G1) were identified as being more specific for USUV E protein DIII than for the WNV protein; in contrast, scFv A9 recognized both USUV and WNV E proteins DIII. The sequences of the resulting scFv are shown in Figure 3.

**FIGURE 4:**
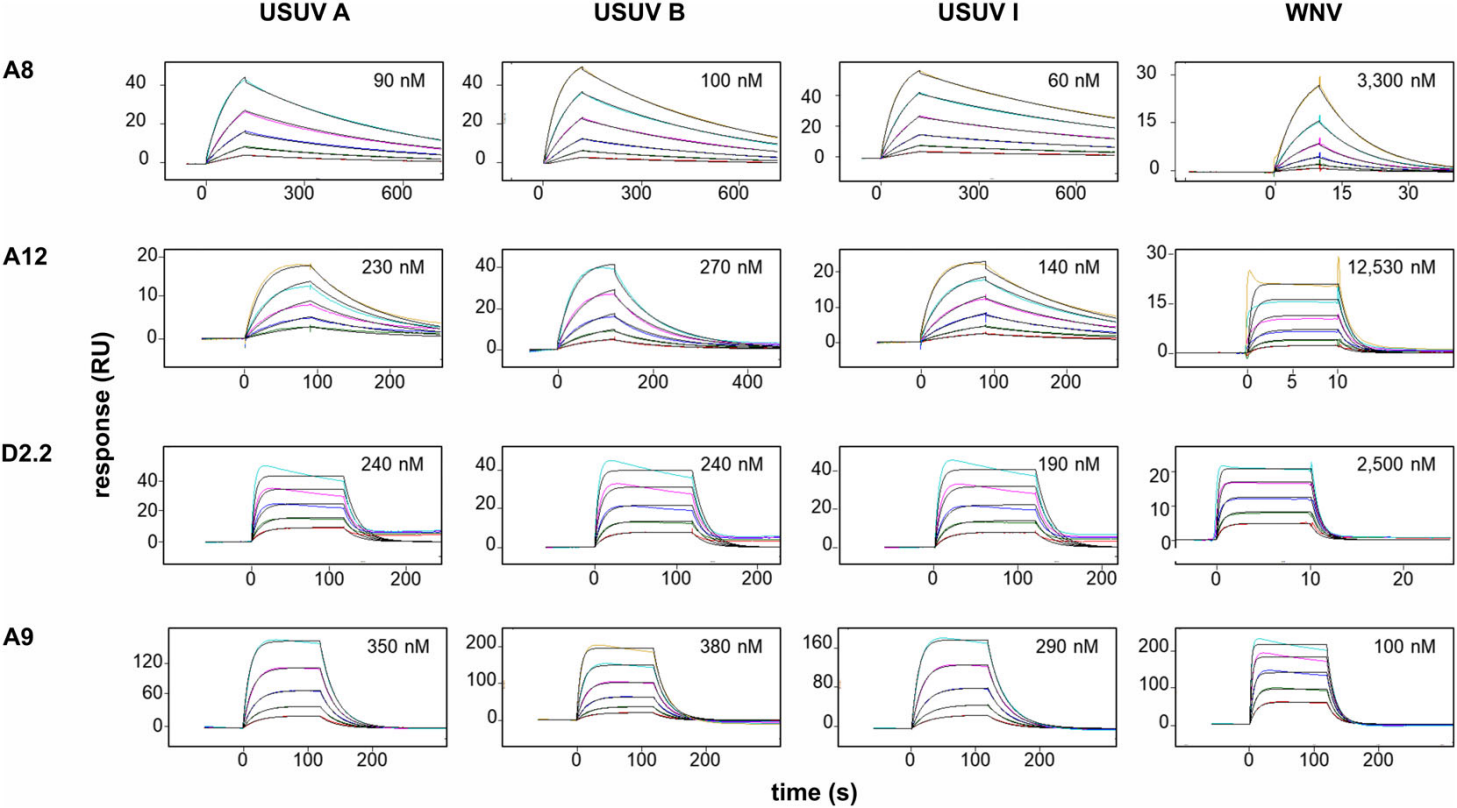
Responses and kinetic fits of scFvs and E protein DIII from USUV and WNV. The responses collected for DIII/scFv interactions in SPR measurements were analyzed with the kinetic model. A fit of the 1:1 interaction overlaid atop each data set fit. For each measurement, the ligand (scFv) was immobilized via its His‐tag and the binding to seven two‐fold dilutions of the analyte (E protein DIII) was measured. All data points were measured in duplicates except for the interaction with the antibody A8, and the interactions of WNV E protein DIII with A12 and D2.2

To further characterize the five scFv described above, we used SPR to measure their binding kinetics to the various DIII proteins and thus derive their binding affinities. The SPR data are shown in Figure 4; the observed kinetic and binding parameters are given in Tables 5 and 6. Data was obtained from the scFvs A8, A12, D2.2, and A9. Despite sharing similar sequences, all four antibodies showed distinct association and dissociation kinetics (Table 5). Both A9 and D2.2 associated comparatively rapidly with the antigens but also dissociated rapidly; in contrast, the reverse situation was observed with antibodies A8 and A12. The determination of the kinetics for the scFv G1 was, however, not possible as the immobilization on the chip of this scFv was instable. This may have resulted from an inaccessibility of the His‐tag and/or partial denaturation of the protein either after purification or under the assay conditions.

Table 6 summarizes the affinity constants for all scFvs calculated from the kinetic parameters in Table 5. A12 was the most specific binder with about 60 times higher affinity to USUV (*K*_D_ 0.14–0.30 μM) than to WNV (*K*_D_ 12.53 μM). A8 had however the strongest affinity for a DIII protein with a *K*_D_ of 0.06–0.10 μM to USUV DIII; the affinity to WNV DIII was about 40 times lower (*K*_D_ 3.29 μM). D2.2 could discern DIII from the two viruses with a 10‐fold higher affinity towards USUV (*K*_D_ 0.19–0.24 μM). A9, the cross‐reactive scFv, bound WNV and USUV DIII at a *K*_D_ of around 0.10 μM and 0.29–0.38 μM, respectively (Table 6).

We examined the sequences of the respective scFv to see whether there was any correlation between the sequences and the affinity of the encoded scFv. The strongest USUV DIII binding scFvs shared very similar sequences in the heavy chain region but differed considerably in the light chain sequences, most noticeably in CDR1 and CDR3. Presumably, the difference in their affinities lies in these regions. In contrast, D2.2 and G1 are similar in the light chain but differ greatly in the heavy chain, especially in the heavy chain because of a large deletion in G1. A9, the scFv that recognizes equally well the DIII of USUV and WNV differs most markedly from all the others in the CDR3 regions of both chains.

### 2.4 *USUV‐specific scFvs can detect flavivirus antigens in immunoassays*

**FIGURE 5:**
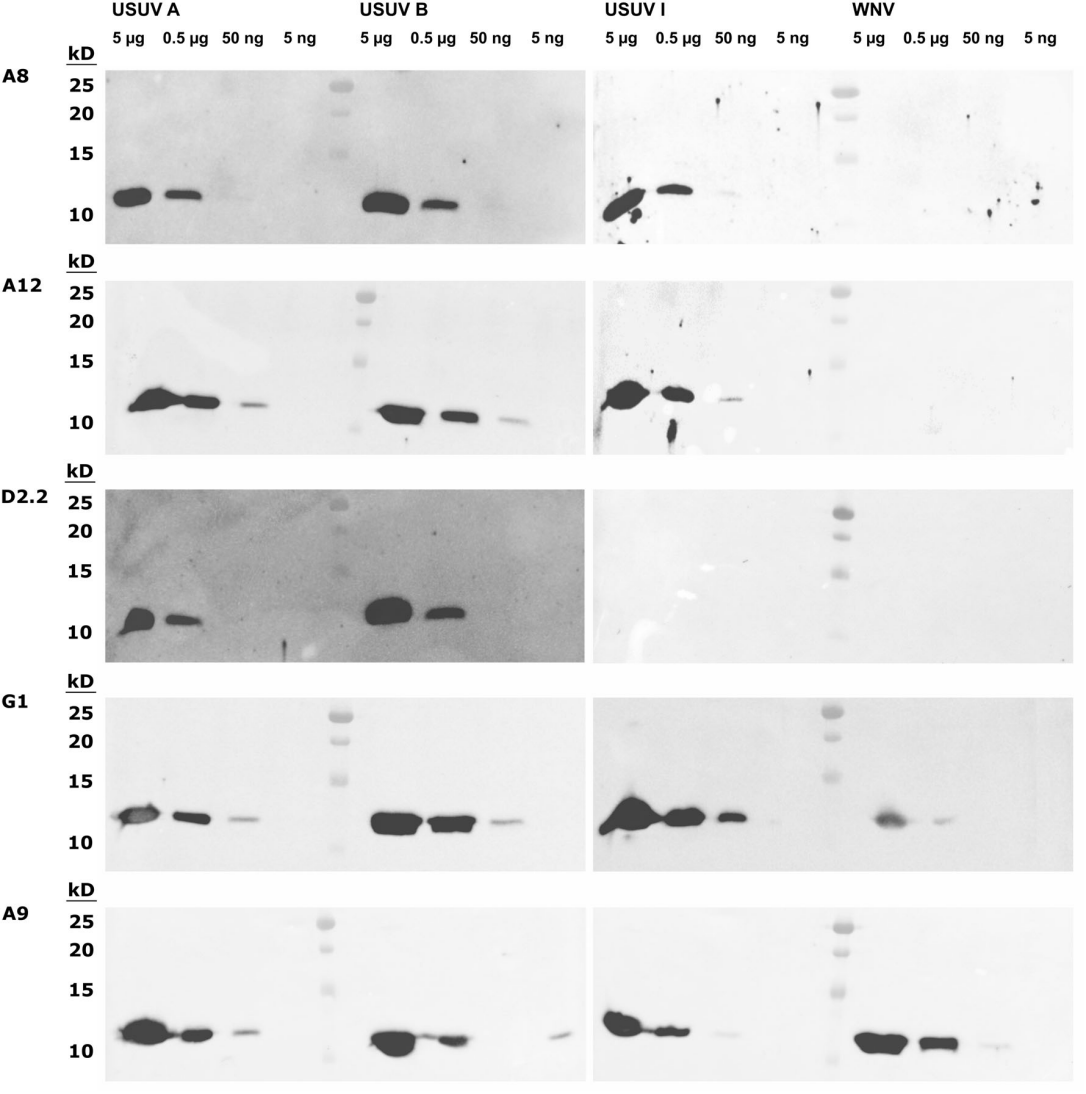
Western blot titrations of scFvs using E protein DIII of USUV and WNV as antigens. Immunoblots of decreasing amounts of USUV E protein DIII from groups A, B, and I as well as of WNV 2741 E protein DIII were incubated with the four scFvs (A8, A12, D2, and A9) at a dilution of 1:2,000, followed by mouse anti‐HA antibodies, and HRP‐conjugated goat anti‐mouse antibodies for visualization

All five scFv (A8, A12, D2.2, G1, and A9) were expressed, purified, and tested for binding to DIII from WNV and USUV group A, B, and I in western blots. The results in Figure 5 show that A8 and A12 clearly bound to all three USUV DIII variants down to 0.5 μg. In the case of A12, faint bands at 50 ng of antigen could be detected. However, neither A8 nor A12 cross‐reacted with up to 5 μg of WNV DIII. The scFv antibody D2.2 bound specifically to USUV DIII group A and B but not to group I and WNV DIII. Only the scFv antibody G1 that could not be evaluated in SPR measurements previously gave strong bands for all USUV DIII groups down to 50 ng. However, faint bands for WNV DIII were also visible. The cross‐reactive A9 scFv antibody bound all USUV DIII variants as well as WNV DIII at relatively comparable levels.

**FIGURE 6:**
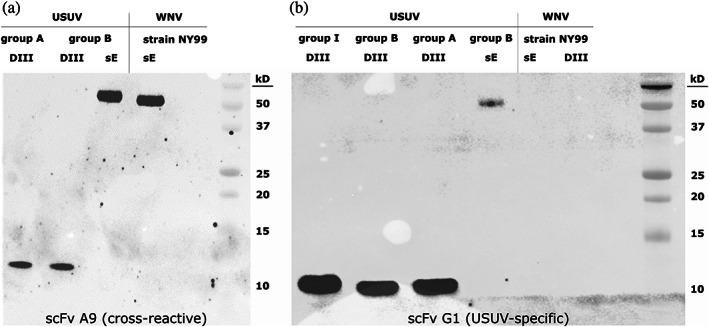
Western blots with E protein DIII and sE of USUV and WNV as antigens. Western blots of DIII from the E protein and sE (the soluble ectodomain of protein E) proteins of USUV and WNV were incubated with the scFvs A9 and G1 at a dilution of 1:2,000, followed by an incubation with mouse anti‐HA antibodies and HRP‐conjugated goat anti‐mouse antibodies. (a) The cross‐reactive scFv A9 reacted with DIII from the E protein and sE of both USUV and WNV. 0.5 μg of antigen per well was loaded on a 15% SDS gel and incubated with 5 μg of scFv in 10 ml of ×1 PBST. (b) 0.5 μg of E protein DIII and 0.25 μg of sE was loaded per well and incubated with 5 μg of the specific scFv G1 in 10 ml ×1 PBST

To further confirm that the antibodies also bind the ectodomain of the E protein and not only the isolated DIII, analogous western blots were conducted. Therefore, the whole ectodomains of the E protein of USUV Bologna‐2009 and WNV NY99 were expressed as soluble E (sE) proteins. Indeed, the cross‐reactive antibody A9 recognized the sE proteins of USUV Bologna‐2009 and WNV NY99 and the E protein DIII from both USUV group A and group B (Figure 6a). In a next step, we tested the specific scFv on E protein DIII and sE of both USUV and WNV. Figure 6b shows that G1 bound to all three USUV E protein DIII groups as well as to the sE of USUV but not to either protein version of WNV.

### 2.5 *Virus ELISA and neutralization*

**FIGURE 7:**
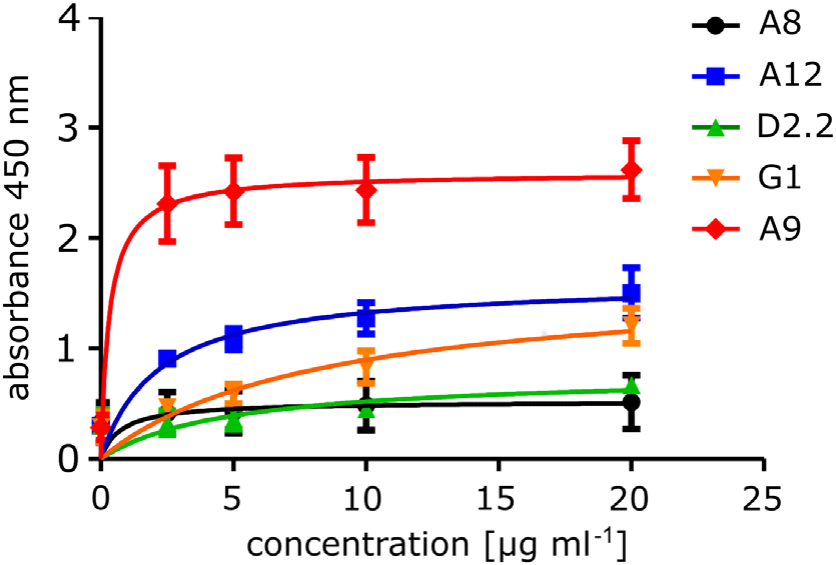
Virus ELISA binding assay on USUV Vienna‐2001. A virus ELISA was performed with all five scFvs (A8, A12, D2.2, G1, A9) in two‐fold dilution steps (2.5–20 μg ml^−**1**^). An anti‐His antibody conjugated with horse‐radish peroxidase was added. The data was obtained from two independent experiments performed in duplicate (*n* = 2)

Based on these findings, we proceeded to test the scFv antibodies on the Vienna‐2001 strain of the USUV with a virus ELISA, using an anti‐His antibody to be able to compare the results to those of the SPR. As can be seen in Figure 7, all scFv bound the whole virus. A9 whose dissociation rate in SPR was the highest (unpublished work), surprisingly bound the whole virus the strongest. It appears that the cross‐reactive A9 is more potent at binding to DIII in the context of the whole virus than the specific scFvs. The most promising scFv A8 with the lowest *K*_D_ for USUV yielded the lowest absorbance values in this assay. The virus seemed to be saturated by both antibodies A8 and A9 at a concentration below 5 μg ml^−1^ while A12 and D2.2 approached a saturation only at around 20 μg ml^−1^. In line with the putative partial unfolding from earlier SPR measurements, G1 did not saturate the virus even at the highest concentration tested (20 μg ml^−1^).

All purified scFv antibodies were screened in neutralization tests as described in the materials and methods. However, all scFv preparations failed to neutralize the USUV strains Vienna‐2001 and SAAR as well as the WNV strain NY‐1999 in concentrations up to 1 μM (data not shown).

## 3 DISCUSSION AND CONCLUSION

USUV has recently emerged in Europe and caused several human infections resulting in mild to severe disease symptoms. As the incidence of USUV is currently still low, the presence of USUV has often been overlooked for the benefit of more widespread viruses such as WNV, DENV, and ZIKV. Hence, there is little information available on USUV, including its geographic spread, circulation patterns, seasonality, etiology, and pathogenesis. Furthermore, extensive cross‐reactivity among flaviviruses complicates attribution of infections to a particular virus.

As an approach to developing antibodies specific for USUV, we immunized three chickens each with a different combination of USUV A and USUV I and generated a phage display library from the mRNA of the spleens of these chickens. Phage display libraries have an important advantage in that they are not influenced by antigen specificity and thus should yield a broad spectrum of antibody specificity. In addition, this approach has only recently been used to generate scFvs against Zika virus E protein. However, the reactivity against other flaviviruses was not investigated.^30^ scFvs reactive against USUV DIII were indeed found by panning of the phage displayed libraries of chicken scFvs. To reduce cross‐reaction with WNV, the resulting panel of 380 antibody candidates was subjected to a monoclonal ELISA so that phage presenting USUV‐specific antibodies on their surface were identified. Four USUV‐specific as well as one cross‐reactive scFv for WNV were obtained in this way. The expressed and purified scFv were further characterized by ELISAs and SPR. The SPR results quantified the differences in the affinity of the scFvs and identified the scFvs A12 and A8 as having 60‐ and 40‐times higher affinity to USUV than WNV, respectively. The differences in the scFv specificity appeared to lie to a large extent in the amino acid sequences found in the CDR3 of both the heavy and the light chains (Figure 3). A similar importance of the CDR3 in antibody specificity in general^31^ and towards the DIII epitope of dengue virus^32^ have been mentioned previously.

We further examined the antibodies regarding their potential use in standard immunodiagnostic applications. First, we wanted to ensure that the scFv antibodies are applicable for western blots in which the antigens are denatured and linearized. Indeed, the binding behavior of the antibodies was consistent with the SPR results in which the cross‐reactive A9 binds to all DIII protein versions from both USUV and WNV. In contrast, the scFvs A8, A12, and D2.2 did not show visible binding to WNV DIII in western blots which is in line with the SPR measurements. Only G1 exhibited low binding affinity to WNV DIII which agrees well with earlier ELISA results. From preceding SPR and ELISA assays, D2.2 should bind DIII from group I in a similar manner to group A and B. However, on western blots, binding to group I DIII could not be observed. This might be due to a discontinuous binding interface of the two binding partners in which the interaction is not strong enough when the antigen is linearized.

We proceeded to successfully test two antibodies on the sE protein of WNV NY99 and USUV Bologna‐2009 on western blots. Based on these findings we examined the binding affinity of the scFv antibodies in whole virus ELISA. All five scFv antibodies bound USUV, with the cross‐reactive A9 eliciting a much higher absorbance than the other specific scFv antibodies. A8, which had the lowest *K*_D_ in SPR experiments, yielded the lowest absorbance values. Comparing data collected in this study, we concluded that A12 shows the best potential as a specific antibody for USUV: A12 had the highest specificity for USUV as demonstrated by the SPR results as well as by western blot titrations and was the best specific binder as shown in virus ELISAs. Nevertheless, all antibodies failed to neutralize either USUV or WNV.

Taken together, the recombinant USUV DIII proteins are a potential target for vaccine development or used as a diagnostic antigen tool in western blot and ELISA assays. Furthermore, this study solidifies the potential of the analyzed scFv antibodies as tools in detection assays for USUV as these antibodies have been found to be effective in distinguishing USUV from the cross‐reactive and co‐circulating WNV. Further, a comprehensive study on cross‐reactivity with other flaviviruses should be conducted to further validate the specificity of these scFv antibodies for future use in diagnostic tests in other geographic areas.

Additionally, their scFv character provides an excellent platform for prospective optimization via directed evolution or specific mutation based on structural data to improve specificity and even neutralizing activity of an scFv. Besides this optimization, a conversion of scFv to either Fab or IgG versions could also enhance their usefulness. It has previously been described that a conversion of IgGs to scFv resulted in a loss of neutralizing function.^33^ Therefore, we hypothesize that a conversion from scFv to IgG might beneficially influence the neutralizing potency. Analogously, a humanization of the scFv might change their overall behavior; it remains to be determined whether the scFvs will retain antigen specificity, avidity, and neutralizing activity upon conversion or humanization.

## 4 MATERIALS AND METHODS

### 4.1 *Expression of antigens DIII and E protein*

The DIII proteins were expressed as described previously (unpublished work). In brief, plasmids encoding for WNV DIII and USUV DIII group A, B, and I were transformed into competent *E. coli* BL21 *and* SoluBL21 cells (Novagen) and grown in LB‐amp at 37°C. The cultures were induced with 0.5 mM IPTG after reaching an OD_600_ of 0.6 and grown for another 4 hr at 23°C. The proteins were denatured with 5 M urea and refolded from inclusion bodies in a two‐step dialysis (3–0 M urea). The proteins were further purified via size exclusion chromatography and filtered.

Soluble E (sE) proteins (containing the whole ectodomain of E) of the Bologna‐2009 strain of USUV and the NY‐1999 strain of WNV expressed in Drosophila S2 cells were kindly provided by Prof. Karin Stiasny and Lena Roßbacher.^34^

### 4.2 *Raising anti‐USUV antibodies in *Gallus gallus domesticus**

All animal procedures were approved by the Animal Care and Use Committee of the Medical University of Vienna (Ethics approval no.: 66.016/0014‐II/3b/2011), and all these procedures^35^ were conducted according to the guidelines established by this committee.

One White Leghorn chicken and two Brown Leghorn chickens (*Gallus gallus domesticus*) were immunized with DIII from the E protein of USUV group A and group I. One Brown Leghorn was immunized with alternating antigens, while the other chickens were administered a 1 to 1 ratio of both antigens. Priming immunization was performed by mixing one part of 200 μg of the respective antigen in water with one part of Complete Freund's Adjuvant (CFA) (F5881 Sigma, Austria) in a total volume of 600 μl. Injections were administered intramuscularly into the breast muscle. For boosting another 200 μl of the antigens were administered with Incomplete Freund's Adjuvant (IFA) (F5506 Sigma, Austria) at a ratio of 1 to 1. A total of five boosts were performed at two‐week intervals. After the antibody titers and the specificity of the raised IgY in the egg yolk were confirmed by ELISA, the animals were sacrificed, and the spleen and bone marrow were harvested for antibody library construction.

### 4.3 *IgY titering via ELISA*

For tracking and quantification of the immune response in the immunized specimen an IgY titering was carried out. Egg yolk of five eggs was collected, pooled and quantified. Saline phosphate buffer was added to facilitate centrifugation for separation of the lipophile form the watery phase by precipitation with 10.5% PEG 8000. The supernatant was subjected to 42% PEG 8000 for precipitation of the IgY. The pellet was re‐dissolved in 4 M ammonium sulphate and recentrifuged. The pellet was dissolved and dialyzed against phosphate buffer (pH 7.5), aliquoted, and stored at −20°C. For the ELISA, 0.1 μg of antigen in a volume of 50 μl was coated onto 96‐well polystyrene microtiter plates (Corning, USA) in carbonate coating buffer overnight at 4°C followed by blocking with 5% milk in PBS. The IgY preparations were subjected to twofold serial dilutions in PBS supplemented with 5% milk and incubated in the coated microtiter plates. The plates were washed five times with PBST (0.05% Tween‐20) and probed with 1:30,000 rabbit anti‐chicken IgY HRP conjugate (Sigma, Austria) for 1 hr at 37°C. After five more washes with PBST, colorimetric development was performed by addition of ABTS substrate (Roche, Switzerland) and the reaction was stopped with 1 M sulfuric acid after 45 min. The absorbance was measured at 405 and 416 nm.

### 4.4 *Library construction and amplification*

The phage display antibody library was constructed as previously described.^36^ The RNA from spleenocytes was extracted using innuPREP RNA Mini Kit 2.0 (analytikjena, Germany) and the complementary DNA (cDNA) was synthesized using the Superscript III Reverse Transcription Kit (Invitrogen, USA) according to the manufacturer's instructions. For PCR amplification of the V gene rearrangements using Q5 polymerase, one *V*_H_ amplification and one *V*_λ_ amplification per chicken were performed according to protocol 9.9 from the CSHL Phage Display Manual using the primer pairs CSCVHo‐FL and CSCG‐B, and CSCVK and CKJo‐B, respectively^36^ (see Table 1). The PCR products of each *V*_H_ and *V*_λ_ amplification were pooled and purified via gel extraction. For the overlap extension PCR, the appropriate first‐round products were mixed in equal ratios to generate the overlap product using the primer pair CSC‐F and CSC‐B in equimolar concentrations, which link the *V*_*λ*_ to *V*_H_ with a long linker of the amino acid sequence GQSSRSSGGGGSSGGGG. The ca. 750 bp long PCR products were pooled and purified. The purified scFv amplicons and the pComb3XSS phagemid vector were digested with SfiI (NEB, USA), gel purified and ligated using T4 ligase (Invitrogen, USA), which extends the scFv by a 6xHis‐Tag and an HA‐Tag. The ligated product was ethanol precipitated and electroporated into electrocompetent *E. coli* XL‐1 Blue. The library size was determined by plating out the electroporated cells onto LB agar containing 100 μg ml^−1^ ampicillin. The library quality was assessed by library size determination, a colony PCR screening, and diversity determination by DNA fingerprinting with BstNI digestion. For amplification, the library stock was inoculated into 5 ml SB medium and grown at 37°C at 250 rpm. After 1 hr, 10 ml of prewarmed SB medium containing 20 μg ml^−1^ ampicillin and 10 μg ml^−1^ tetracycline was added and shaken at 250 rpm and 37°C. After 1 hr, the ampicillin concentration was raised to 50 μg ml^−1^. Phagemids were rescued by infection of the XL‐1 Blue culture with 10^12^ pfu ml^−1^ M13K07 helper phage (Life Technologies) and adding SB medium to 200 ml containing 50 μg ml^−1^ ampicillin and 10 μg ml^−1^ tetracycline at 300 rpm and 37°C. After 2 hr, 70 μg ml^−1^ kanamycin was added, and the culture was incubated at 37°C overnight with shaking at 300 rpm for production of phage particles. Phage were purified from the culture supernatant by precipitation with 1/5 (vol./vol.) of 20% (wt./vol.) PEG‐6000 and 2.5 M NaCl on ice for 30 min. Precipitated phage was resuspended in 2 ml of 1% (wt./vol.) BSA in TBS, filtered and used directly for panning. The remaining stock was provided with 20% glycerol before aliquoting and storage at −80°C.

**TABLE 1:** Primers used for PCR amplification of antibody variable regions and overlap extension PCR, and for sequencing

	Sequence 5′‐3′
V_H_ primers	
CSCVHo‐FL (sense)	GGT CAG TCC TCT AGA TCT TCC GC GGT GGT GGC AGC TCC GGT GGT GGC GGT TCC GCC GTG ACG TTG GAC GAG
CSCG‐B (reverse)	CTG GCC GCC CTG GCC ACT AGT GGA GGA GAC GAT GAC TTC GGT CC
V_λ_ primers	
CSCVK (sense)	GTG GCC CAG GCG GCC CTG ACT CAG CCG TCC TCG GTG TC
CKJo‐B (reverse)	GGA AGA TCT AGA GGA CTG ACC TAG GAC GGT CAG G
Overlap extension primers	
CSC‐F (sense)	GAG GAG GAG GAG GAG GAG GTG GCC CAG GCG GCC CTG ACT CAG
CKJo‐B (reverse)	GAG GAG GAG GAG GAG GAG GAG CTG GCC GGC CTG GCC ACT AGT GGA GG
Sequencing	
Ompseq	AAG ACA GCT ATC GCG ATT GCA G
pCombrev	AAA ATC ACC GGA ACC AGA GC

*Note*: CSCVHo‐FL and CKJo‐B include the long linker sequence tail, while CSCG‐B and CSCVK add the SfiI site.

### 4.5 *Panning and phagemid rescue*

The panning and phage amplification was performed similarly to previously described protocols.^36^, ^37^ For positive selection panning, ELISA plates (Nunc MaxiSorp, Thermo Fisher) were coated with 50 μl of the antigen (round 1 and 2, 20 μg ml^−1^; round 3 and 4, 10 μg ml^−1^) in carbonate coating buffer (0.1 M sodium bicarbonate, pH 9.6) at 4°C overnight and blocked with 150 μl of 3% (wt./vol.) BSA in PBS for 1 h at room temperature. Input phage were about 10^12^ pfu in 200 μl per well and per round of panning. The wells were washed with 150 μl of 0.5% PBST to eliminate unbound phage. Specifically, bound phage was eluted with 200 μl 100 mM glycine‐HCl (pH 2.2) for 10 min and immediately neutralized with 2 M Tris (pH 9.0). Neutralized phage was reamplified in exponentially growing *E. coli* XL‐1 Blue and used for subsequent rounds of panning and phage ELISA. Four rounds of panning with increasing stringency were carried out (see Table 2). Subtractive panning was not included due to the high sequence and structure identity of the E protein DIII from WNV and USUV.

**TABLE 2:** Conditions used for subsequent rounds of panning to enrich clones

	First round	Second round	Third round	Fourth round
Phage input (cfu)	Phage library stock (6.6 × 10^12^)	Amplified first round phage (3.1 × 10^12^)	Amplified second round phage (1.1 × 10^13^)	Amplified third round phage (2.5 × 10^12^)
Phage output (cfu)	4.7 × 10^6^	2.3 × 10^6^	2.9 × 10^4^	2.9 × 10^7^
Antigen USUV	Group A	Group B	Group A	Group B
Antigen (μg ml^−1^)	20	20	10	10
Number of wells	4	4	4	2
Number of washes	5	10	15	15

### 4.6 *Polyclonal and monoclonal phage ELISA*

A polyclonal ELISA was performed to confirm enrichment for scFv‐phage binders over the rounds of panning. Phage‐containing supernatants from the rounds of panning were incubated in antigen‐coated microtiter plates (50 μl of 5 μg ml^−1^ in coating buffer) after reamplification. BSA and PBS coated wells served as negative controls. Plates were washed five times with PBST and probed with 1:5,000 mouse HRP‐anti‐M13 monoclonal antibody in 5% milk in PBST (160,325, GE Healthcare, UK). After a final wash with PBST, colorimetric development was performed by addition of 50 μl TMB substrate per well, reaction stopped with 12.5 μl of 3 M HCl and the absorbance was measured at 450 nm (Tecan plate reader).

For monoclonal phage ELISA, library clones each from the second, third and fourth rounds of panning were individually rescued. Therefore, a total of 380 colonies were inoculated into 500 μl of medium supplemented with 100 μg ml^−1^ ampicillin, 2% glucose and 10 μg ml^−1^ tetracycline, and grown overnight at 37°C and 800 rpm as seed cultures. Five microliters of overnight seed culture were inoculated individually into 500 μl fresh medium, grown at 37°C and 800 rpm to an OD of approximately 0.5, and infected at 37°C and 800 rpm for 30s with 1.5 × 10^9^ M13K07 helper phage and grown for an additional 30 min at 37°C without shaking. The medium was replaced with 500 μl of medium with 50 μg ml^−1^ kanamycin added and grown at 30°C and 800 rpm overnight for production of phage particles.

Small‐scale phage rescue supernatant from individual clones was diluted by one fourth with 5% milk in PBST and 100 μl of this dilution were incubated on antigen‐coated microtiter plates. Washing, incubation with the antibody and absorbance measurements were carried out as in the polyclonal phage ELISA. Clones producing absorbance values higher than two‐fold background levels on captured USUV E protein DIII and two‐fold higher than on captured WNV E protein DIII were considered to be positive and were selected for further characterization.

### 4.7 *Characterization and expression of scFv antibody fragments*

Plasmids from clones that showed specific affinity to USUV were extracted, purified and subjected to fingerprinting by BstNI before being sequenced with the primers ompseq and pCombrev (see Table 1). ScFv‐coding DNA sequences were amplified by PCR and digested with the enzyme BstNI and analyzed on a 2% agarose gel electrophoresis. Clones producing visually distinct restriction patterns were characterized separately. Amino acid residue alignments and analyses were performed using Clustal Omega^38^ and MEGAX.^39^ CDR identification was performed based on the IMGT/V‐QUEST.^40^, ^41^ The best candidates from the monoclonal ELISA were expressed as previously described.^36^ In short, the phagemid DNA in pComb3XSS was transformed into chemically competent *E. coli* TOP10. One liter of culture was grown in SB medium supplemented with 50 μg ml^−1^ ampicillin and 20 mM MgCl_2_ at 37°C and 255 rpm for 8 hr. The culture was induced with 1 mM IPTG and grown overnight at 37°C. The cell pellets were harvested and resuspended with PBS supplemented with 200 μM PMSF (10 ml per 1 L expression culture). The resuspended pellets were lysed by sonication on ice for 180 s, pulsing at 50% duty cycle (Tekmar sonic disruptor) and the cellular debris was removed by repeated centrifugation at the maximum speed. The expressed 6‐His‐tagged scFv was purified from the lysed pellet fraction and the expression supernatant using IMAC (HisTrap FF, GE Healthcare, USA) by batch purification according to the manufacturer's recommendations using a wash buffer (50 mM NaH_2_PO_4_, 300 mM NaCl, 20 mM imidazole, pH 8.0) and an elution buffer (wash buffer with 250 mM imidazole). The purified scFv antibodies were dialyzed against PBS, sterile filtered, monitored by 15% SDS‐PAGE quantified by Bradford protein concentration assay and stored at −20°C until further use.

### 4.8 *Western blots*

The protein immunoblots were carried out according to the standard protocol using in‐house produced mouse anti‐HA antibodies and mouse anti‐His antibodies as primary antibodies and goat HRP‐anti‐mouse antibodies as secondary antibodies.

### 4.9 *Functional characterization of soluble scFv by indirect ELISA*

An indirect scFv ELISA was developed for screening of the expressed scFv antibodies for USUV E protein DIII specificity. Therefore, ELISA plates (Nunc MaxiSorp, Thermo Fisher) were coated with 50 μl of antigen at 5 μg ml^−1^ in carbonate coating buffer and blocked with 3% milk in PBS. 150 μl of each antibody dilution (5 μg ml^−1^) was incubated with the antigen for 2 hr at room temperature. After five times washing with PBST, the primary mouse antibody against the HA‐tag of the scFv was diluted 1:2,000 and incubated for 1 hr. The secondary HRP‐anti mouse antibody was diluted 1:5,000 and incubated for another 1 hr at room temperature. After a final wash, colorimetric detection was performed by addition of 50 μl ABTS solution (Roche), the reaction was stopped by adding 1 M sulfuric acid, and the absorbance was measured at 405 nm.

### 4.10 *Surface plasmon resonance analysis of scFvs*

Surface plasmon resonance (SPR) experiments were performed on a Biacore T200 instrument (GE Healthcare). As a ligand, the scFv antibodies were immobilized while the antigen (E protein DIII) served as the analyte. Their properties are listed in Table 3. The scFv antibodies (5 μg ml^−1^ in 1X PBS pH 7.4; with exception of scFv A8 at a concentration of 15 μg ml^−1^) were immobilized as ligands on flow cell 2 (ligand densities: A8 ~ 270 RU; A12 ~ 330 and 100 RU; D2 ~ 210 and 80 RU; A9 ~ 890 and 160 RU), on a commercially available CM5 chip (GE Healthcare) using the His Capture Kit according to the manufacturer's protocol (GE Healthcare). In short, the flow cell was activated with EDC/NHS and excess reactive carboxyl groups were blocked with ethanolamine. Flow cell 1 was activated and blocked in the same way and served as reference surface without any ligand bound. Multi‐cycle kinetics (MCK) experiments were performed at 25°C using two‐fold dilution series of the DIII protein variants of USUV (31–2,000 nM) and WNV (31–2,000 nM and 100–32,000 nM where applicable). 1X PBS supplemented with 0.05% Tween 20 was used as running buffer. Double referencing was accomplished by subtracting the signal from injections of running buffer. The flow rate was set to 30 μl min^−1^, contact time with analyte varied between 10 and 120 s, dissociation was monitored for 20–600 s (see Table 4). Complete removal of captured ligand for regeneration of the covalently coupled anti‐His antibodies was achieved by one injection of 10 mM glycine‐HCl pH 1.5 (60 s, 30 μl min^−1^) followed by four injections of 10 mM NaOH (30 s each, 30 μl min^−1^) after every cycle. The integrity of the various ligands within one titration experiment was ensured by testing reproducibility of the 250 nM analyte binding curve after application of the highest analyte concentration. Data were analyzed with the Biacore Evaluation Software version 3.1 (GE Healthcare). To determine the dissociation constant *K*_D_, steady‐state response units were plotted against analyte concentrations and the data were fitted to a steady‐state affinity model (Tables 5 and 6).

**TABLE 3:** Identity, source and molecular weight of ligand and analyte

	PDB ID	Number of amino acids	Molecular weight [kDa]	Source	Tags
Ligand (scFv)					
A8	–	276	30.5	*E. coli* TOP10	His, HA
A12	–	279	30.8	*E. coli* TOP10	His, HA
D2	–	275	30.2	*E. coli* TOP10	His, HA
A9	–	270	29.7	*E. coli* TOP10	His, HA
Analyte (antigen: E protein DIII)					
USUV A	6S92	103	11.0	*E. coli* solu BL21	–
USUV B	6S93	103	11.0	*E. coli* solu BL21	–
USUV I	6S95	103	10.9	*E. coli* solu BL21	–
WNV	2P5P	103	10.9	*E. coli* BL21	–

**TABLE 4:** Parameters used for surface plasmon resonance

			Analyte USUV E protein DIII		Analyte WNV E protein DIII	
Ligand	Concentration (μg ml−1)	Association time (s)	Contact Time (s)	Dissociation time (s)	Contact time (s)	Dissociation time (s)
A8	15	120	120	600	10	30
A12	5	75	90	180	10	20
D2	5	120	60	120	10	15
A9	5	60	120	240	120	240

**TABLE 5:** SPR measurements of scFvs and E protein DIII from USUV and WNV

	USUV E protein DIII group A			USUV E protein DIII group B			USUV E protein DIIIGroup I			WNV E protein DIIINY99 strain		
	*K*_D_ (μM)	Ka (Ms^−1^)	*k*_d_ (s^−1^)	*K*_D_ (μM)	Ka (Ms^−1^)	*k*_d_ (s^−1^)	*K*_D_ (μM)	Ka (Ms^−1^)	*k*_d_ (s^−1^)	*K*_D_ (μM)	Ka (Ms^−1^)	*k*_d_ (s^−1^)
A8	0.09	21,820	0.002	0.10	20,030	0.002	0.06	19,790	0.001	3.29	28,630	0.094
A12	0.21	57,320	0.012	0.30	39,640	0.012	0.14	48,630	0.007	12.53	61,480	0.770
	0.24	42,100	0.010	0.29	34,230	0.010	0.13	46,610	0.006	–	–	–
D2.2	0.21	358,300	0.074	0.23	326,200	0.074	0.19	293,000	0.057	2.48	489,100	1.214
	0.28	278,600	0.078	0.25	272,700	0.069	0.18	337,100	0.061	–	–	–
A9	0.35	118,800	0.041	0.41	152,000	0.062	0.29	170,500	0.050	0.10	1,039,000	0.105
	0.31	170,100	0.053	0.36	165,500	0.059	0.30	167,700	0.048	0.10	969,600	0.094

**TABLE 6:** Summary of SPR measurements of scFvs and DIII from USUV and WNV

*K*_D_ (μM)	USUV E protein DIII group A	USUV E protein DIII group B	USUV E protein DIII group I	WNV E protein DIIINY99 strain	WNV DIII to USUV DIII group A
A8	0.09	0.10	0.06	3.29	36.6
A12	0.22	0.30	0.14	12.53	57.0
D2.2	0.24	0.24	0.19	2.48	10.3
A9	0.33	0.38	0.29	0.10	0.3

### 4.11 *Virus ELISA*

The expanded virus from C6/36 cells was precipitated with 8% (wt./vol.) PEG8000 at 4°C overnight, spun down at 14,300 *g* for 1 hr, and resuspended in NTE buffer (12 mM Tris–HCl, pH 8.0, 120 mM NaCl, 1 mM EDTA). The diluted virus suspension (8.1 × 10^6^ pfu in 50 μl) was coated onto 96‐well ELISA plates in volumes of 50 μl at 4°C overnight. The next day, the plate was blocked with 5% BSA at room temperature for 2 hr, followed by repeated washing with 0.1% PBST, and 50 μl scFv antibody preparations were added. After 1 hr of incubation at 37°C, an HRP‐conjugated mouse anti‐His antibody (Thermofisher MA1‐80218) was added and incubated for another hour. Following washing, TMD solution was used for detection and absorbance was measured at 450 nm. Each virus‐antibody reaction was carried out twice (*n* = 2) in duplicates on different days. On each day, all reactions were performed on one plate.

### 4.12 *Neutralization assays*

WNV and USUV neutralization tests were performed as described previously.^42^ Briefly, duplicates of serial two‐fold dilutions of scFv antibodies (starting concentration 1 μM were incubated with 30–60 TCID50 (50% tissue culture infective dose) of WNV strain NY99 or USUV strain SAAR for 1 hr at 37°C. Vero cells (ECACC) were added and incubation was continued for 4–6 days at 37°C. For USUV strain Vienna‐2001, C6/36 cells (ATCC) were added and incubated for 3–4 days at 37°C. Presence of virus in the supernatant was assessed by the occurrence of cytopathic effects.

## AUTHOR CONTRIBUTIONS

**Amelie Schoenenwald:** Conceptualization; data curation; investigation; methodology; project administration; validation; visualization; writing‐original draft; writing‐review and editing. **Chin Piaw Gwee:** Investigation; writing‐review and editing. **Karin Stiasny:** Investigation; writing‐review and editing. **Marcela Hermann:** Investigation. **Subhash Vasudevan:** Supervision; writing‐review and editing.

## Supporting information


**Data S1** Supporting Information
