# Supplementary material for: Development and characterization of specific anti‐Usutu virus chicken‐derived single chain variable fragment antibodies
Source: Protein Sci. 2020 Sep 4;29(11):2175–88. doi: 10.1002/pro.3937 (PMC7586906; doi:10.1002/pro.3937)

SUPPLEMENTARY MATERIAL

Supplementary Figure 1: Chicken IgY are reactive to USUV and WNV E protein DIII in ELISA.

Chicken IgY isolated from egg yolk from the first eggs laid by the chickens after the initial immunization (pre-immune, 5 weeks after first immunization) and after the final immunization (immune, 12 weeks after first immunization) were analyzed in ELISA for reactivity at a 200-fold dilution against E protein DIII from USUV groups A and I and from that of WNV. Reactivity with BSA served as a control. Results are summarized from three independent experiments (n=3) with triplicates each. Error bars represent the calculated standard error.


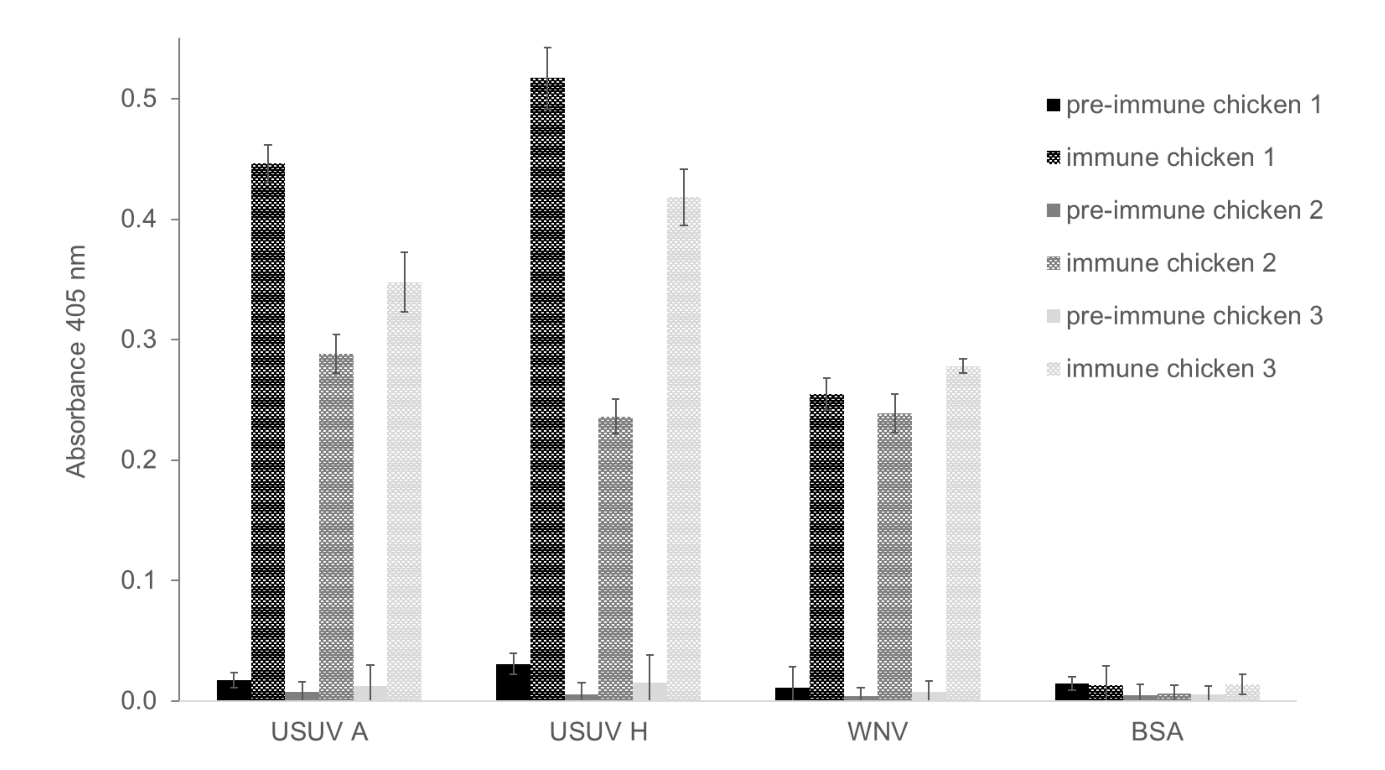


Supplementary Figure 2: Analysis of the phagemid DNA of USUV E protein DIII specific bacteriophage by BstNI fingerprinting.

The genes of the scFv candidates are located within an expression cassette of the phagemid vector pComb3XSS. The restriction site 5’--CC/WGG--3’ of BstNI yields a certain number of DNA fragments depending on the individual sequences of the scFv clones. Each clone thus has a distinct band pattern on DNA gels. On this representative gel, 13 USUV-specific clones were tested via BstNI fingerprinting. Subsequent Sanger sequencing confirmed that all 13 clones had unique sequences. The underlined scFv clones highlight the clones that subsequently showed specificity for USUV E protein DIII. Agarose gel percentage: 2%, run at 120 V for 65 min. Load: 8 µl of a 20 µl digest with a 100 bp marker. Selected sizes are indicated in bp on the right.


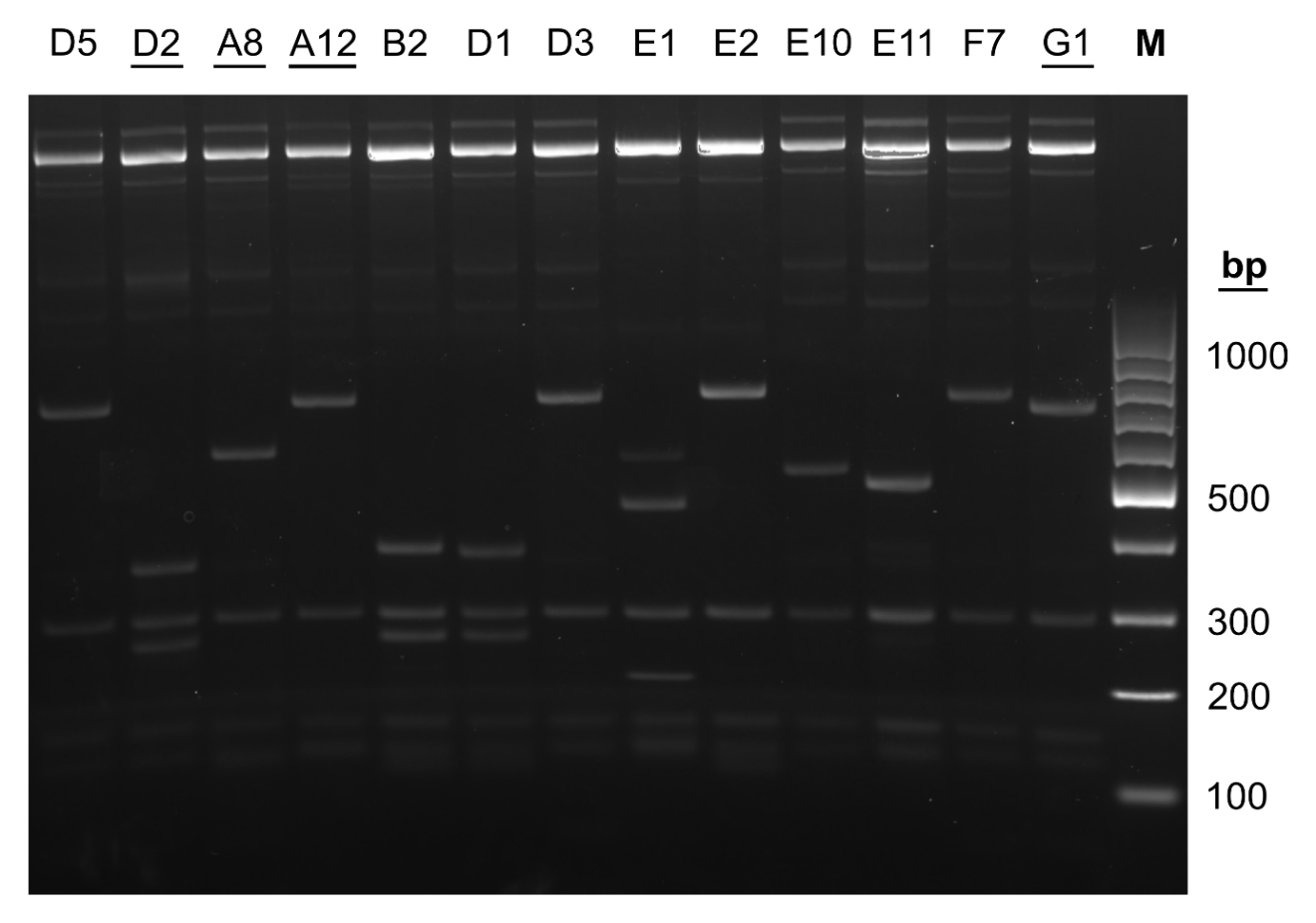

Supplement: Supplementary file 1 — Data S1 Supporting Information [file PRO-29-2175-s001.docx]
